# Molecular epizootiology of porcine reproductive and respiratory syndrome virus in the Xinjiang Uygur Autonomous Region of China

**DOI:** 10.3389/fmicb.2024.1419499

**Published:** 2024-06-26

**Authors:** Junhui Li, Wenjie Gong, Liping Mao, Xiaomei Pan, Qingqing Wu, Yidi Guo, Jianfeng Jiang, Huifen Tang, Yi Zhao, Lanling Cheng, Changchun Tu, Xinglong Yu, Sun He, Wei Zhang

**Affiliations:** ^1^College of Veterinary Medicine, Hunan Agricultural University, Changsha, China; ^2^Tecon Bio-Pharmaceuticals Co. Ltd., Urumqi, China; ^3^College of Veterinary Medicine, Jilin University, Changchun, China; ^4^Changchun Research Veterinary Institute, Chinese Academy of Agricultural Sciences, Changchun, China; ^5^Jiangsu Co-Innovation Center for the Prevention and Control of Important Animal Infectious Diseases and Zoonoses, Yangzhou University, Yangzhou, China

**Keywords:** PRRSV, molecular epizootiology, genetic diversity, recombination, Xinjiang

## Abstract

Rapid evolution of *porcine reproductive and respiratory syndrome virus* (PRRSV) is the bottleneck for effective prevention and control of PRRS. Thus, understanding the prevalence and genetic background of PRRSV strains in swine-producing regions is important for disease prevention and control. However, there is only limited information about the epizootiological situation of PRRS in the Xinjiang Uygur Autonomous Region, China. In this study, blood or lung tissue samples were collected from 1,411 PRRS-suspected weaned pigs from 9 pig farms in Changji, Shihezi, and Wujiaqu cities between 2020 and 2022. The samples were first tested by RT-quantitative PCR, yielding a PRRSV-2 positive rate of 53.6%. Subsequently, 36 PRRSV strains were isolated through initial adaptation in bone marrow-derived macrophages followed by propagation in grivet monkey Marc-145 cells. Furthermore, 28 PRRSV-positive samples and 20 cell-adapted viruses were selected for high-throughput sequencing (HTS) to obtain the entire PRRSV genome sequences. Phylogenetic analysis based on the nucleotide sequences of the ORF5 gene of the PRRSV strains identified in this study grouped into sub-lineages 1.8 and 8.7 the former being the dominant strain currently circulating in Xinjiang. However, the NSP2 proteins of the Xinjiang PRRSV strains shared the same deletion patterns as sub-lineage 1.8 prototype strain NADC30 with the exception of 4 strains carrying 2-3 additional amino acid deletions. Further analysis confirmed that recombination events had occurred in 27 of 37 PRRSVs obtained here with the parental strains belonging to sub-lineages 1.8 and 8.7, lineages 3 and 5, with the recombination events having occurred most frequently in the 5' and 3' termini of ORF1a and 5' terminus of ORF1b.

## 1 Introduction

Porcine reproductive and respiratory syndrome (PRRS) is one of the most devastating diseases for the pig industry worldwide, featuring reproductive failure in pregnant sows and respiratory distress in growing pigs (Neumann et al., 2005). The causative agent, PRRSV (*Porcine reproductive and respiratory syndrome virus*), is an enveloped, positive-sense, single-stranded RNA virus, which belongs to the genus *Betaarterivirus*, subfamily *Variarterivirinae* within the family *Arteriviridae* (Brinton et al., 2021). The PRRSV genome varies in length from 14.9 kb to 15.5 kb and possesses 11 open reading frames (ORFs), with ORF1a and ORF1b accounting for 80% of the viral genome. The polyproteins generated from ORF1a and ORF1b are processed to form 13 nonstructural proteins (NSPs) (Cavanagh, 1997), and the remaining ORFs (ORF2a, 2b, and 3-7) code for minor structural proteins (GP2, GP3, and GP4) and major structural proteins (GP5, M, and N) (Meulenberg et al., 1995; van Dinten et al., 1996). PRRSV NSP2 shows different size polymorphic patterns due to in-frame deletions, which are the unique features for defining the evolutionary characteristics of different lineage viruses (Tong et al., 2007; Brockmeier et al., 2012; van Geelen et al., 2018). GP5 encoded by ORF5 is involved in virion formation through interaction with M protein, and restriction fragment length polymorphism (RFLP) of the ORF5 gene is often used for defining newly emerged PRRSV strains (Shi et al., 2010).

Based on the genetic distances of their viral ORF5 gene, PRRSV strains can be divided into two types: PRRSV-1 (European genotype, prototype strain Lelystad) and PRRSV-2 (North American genotype, prototype strain VR-2332) (Wensvoort et al., 1991; Nelson et al., 1993). PRRSV-1 and PRRSV-2 isolates exhibit substantial genetic and antigenic distances, sharing about 60% nucleotide sequence similarity at the genomic level (Wensvoort et al., 1991). PRRSV-1 and PRRSV-2 strains are both distributed worldwide with the first mainly prevalent in Europe and the latter mainly in the Americas and Asia. Since PRRSV strains are prone to rapid evolution through accumulation of mutations and recombination, extensive genetic diversity is observed within each type. Shi et al. (2010) developed a global PRRSV classification system, based on the genetic diversity of the ORF5 gene, by which PRRSV-1 can be separated into 4 subtypes (I-IV) with subtype I as dominant, and with PRRSV-2 separated into 9 diverse lineages (lineages 1-9). Of these lineages, 7 are found mainly in North America and 2 exclusively in Asia, some of which can be further divided into several sub-lineages, with sub-lineages 1.5, 1.8, and 8.7 currently dominant in China (Dong et al., 2017; Yu et al., 2020).

PRRSV was first documented in North America in 1987 and in Western Europe in 1990 (Collins et al., 1992). The prototype strain of PRRSV-1, Lelystad, was isolated in 1991 in Netherland (Wensvoort et al., 1991). In 1996, PRRSV-2 strain CH-1a belonging to lineage 5 was first isolated in China, but its damage to swine health did not attract much attention until the emergence in 2006 of highly pathogenic PRRSV (HP-PRRSV, sub-lineage 8.7) from a less pathogenic variant (Tian et al., 2007), after which HP-PRRSV quickly spread and became dominant throughout the country, causing considerable economic losses in the swine industry of China. In 2009, a PRRSV-1 strain was first isolated in China, although PRRSV-1 infection here has been traced back to 1999. The following year, strain QYYZ of another lineage (lineage 3) was detected, with viruses of this lineage becoming prevalent in southeast China (Xie et al., 2014). Additionally, virulent PRRSV NADC30 strains (sub-lineage 1.8) had emerged in the United States in 2008 (Brockmeier et al., 2012), and NADC30-like viruses were detected in 2012 in China through imported pigs, becoming dominant in this country (Zhou et al., 2015; Li et al., 2017). More recently, sub-lineage 1.5 strains (representative strain NADC34) emerged in 2014 in USA, showing the restriction fragment length polymorphism (RFLP) 1-7-4 in the ORF5 gene (van Geelen et al., 2018), and were first detected in 2017 in China. NADC34-like PRRSV strains now widespread throughout this country (Xie et al., 2020; Xu et al., 2020).

Accumulated mutations in the viral genomes, especially deletions and recombination, have greatly contributed to the rapid evolution of PRRSV, leading to the emergence of viral variants forming new sub-lineages or lineages that have become the dominant lineages at different times and geographic regions (Xie et al., 2020; Xu et al., 2020). The lineage shifts of PRRSV have major implications for the prevention and control of the disease. While extensive epizootiological investigations of PRRS have been conducted over most of the country, until now only limited information existed about the prevalence of PRRSV infection in the Xinjiang Uygur Autonomous Region. This report summarizes an investigation of the molecular epizootiology of PRRSV using more than one thousand samples collected mainly between 2020 and 2022 from 9 farms in Xinjiang.

## 2 Materials and methods

### 2.1 Clinical samples and detection of PRRSV by real-time quantitative PCR

Samples of lung and blood were collected between 2020 and 2022 from 1,411 aborted pig fetuses or sick pigs with respiratory disease from 9 large-scale pig farms in Changji, Shihezi and Wujiaqu cities of the Xinjiang Uygur Autonomous Region. The lung samples were first homogenized as 10% tissue suspensions and then clarified by centrifugation. The resulting supernatants together with the blood samples were subjected to total RNA extraction using the QIAamp viral RNA kit according to the manufacturer's instructions (Life Technologies, USA). cDNA synthesis and qPCR were performed with the real-time RT-PCR PRRSV Type1 and Type 2 Multiplex RNA test (Hunan Guoce Biotech, China). The reaction was conducted for 40 cycles with reverse transcription at 42°C for 5 min, pre-denaturation at 95°C for 10 s, denaturation at 95°C for 5 s and annealing and extension at 60°C for 35 s. Results were determined by plotting amplification curves of fluorescence signal vs. cycle threshold (Ct) values, with values ≤ 40 considered positive as specified in the product's manual.

### 2.2 *In vitro* adaptation of PRRSV isolates

The 10% tissue suspensions of PRRSV-positive lung samples were clarified and then passed through 0.22 μm syringe filters (Millipore, USA). The filtered supernatants were incubated with bone marrow-derived macrophages (BMDMs) grown in 6-well cell culture plates (Corning Inc., USA) until 80% confluency, then the inocula were replaced with DMEM containing 10% FBS after incubation for 1 h, and the cells were maintained at 37°C in a 5% CO_2_ humidified incubator. Cultures were harvested when cytopathic effects (CPE) were observed in 80% of the cells. Isolated PRRSV strains were passaged 3 times in BMDMs, followed by passage in grivet monkey Marc-145 cells. Viral infections were confirmed by indirect immunofluorescence test (IFA) using 1:100 diluted swine anti-PRRSV serum (VMRD, USA) as the first antibody and 1:200 diluted FITC-conjugated rabbit anti-pig IgG H&L (Sigma, USA) as the secondary antibody.

### 2.3 Genome sequencing of PRRSV strains by HTS

To obtain the full-length PRRSV genome sequence for genetic and evolutionary analysis, meta-transcriptomics (MTT) was carried out as described previously (Jiang et al., 2022). In brief, total RNA was extracted from the clarified aliquots of 10% lung tissue homogenate or blood samples using Trizol (Thermo Fisher Scientific), from which rRNA was removed by Epicenter Ribo-zero™ rRNA Removal Kit. Following precipitation by ethanol, the remaining RNA was resuspended and used for construction of an RNA library using NEBNext^®^ Ultra™ Directional RNA Library Prep Kit according to the manufacturer's instructions. Paired-end (150 bp) sequences of both RNA and DNA libraries were sequenced by the Nova 6000 platform (Illumina, USA). After quality control and removal of reads mapping to the host genome, the remaining reads were *de novo* assembled into contigs using MEGAHIT v1.1.3, and then confirmed through alignment to the NCBI non-redundant nucleotide and protein databases with e-values set to 1 × 10^−10^ and 1 × 10^−4^, respectively.

### 2.4 Sequence comparison and phylogenetic analysis

Analysis of the nucleotide sequence identity of PRRSV genomes or individual genes was conducted with the Megalign program implemented in the Lasergene software package v7.0 (DNAstar). Multiple amino acid sequence alignments of PRRSV ORF5 and nsp2 proteins were conducted with the CLC Sequence Viewer 8.0 (Qiagen, Germany). Phylogenetic analysis based on the nucleotide sequences of ORF5 gene obtained in this study and the corresponding gene sequences of representative PRRSV strains retrieved from GenBank was performed using MEGA v7.0 (Kumar et al., 2016) by the maximum-likelihood method with bootstrap 1,000 replicates. The best fitting substitution model for phylogenetic analysis of ORF5 gene was calculated using MEGA v7.0.

### 2.5 Recombination analysis

Potential recombination events in the PRRSV genomes were detected by RDP4 software (Martin et al., 2015), using 7 different algorithms (RDP, GENECONV, BootScan, MaxChi, Chimera, SiScan, and 3Seq) at default settings. Recombinant events were considered positive when at least 5 algorithms were supported, and breakpoints were verified and visualized by Simplot version 3.5.1.

## 3 Results

### 3.1 Detection and isolation of PRRSV from clinical samples

Real-time RT-PCR results showed that 756 of the 1,411 (53.6%) blood and lung samples analyzed were positive only for PRRSV-2 with 24.6%−83.8% positive rates in each farm (Table 1). PRRSV-1 was not detected. In the 5 swine-producing farms located in Changji, the positive rates of PRRSV in the sick pigs were all >50% in different years between 2020 and 2022 except in samples collected in 2021 in the ZS farm (32.9%). In addition, the positive rates of PRRSV infection in samples from the ZS and DB farms increased in 2022 compared with 2021, but the infection status of PRRS was stable in farms YQ and JM between 2020 and 2022. Detection rates of PRRSV in the collected samples from the 2 farms located in Wujiaqu were relatively low (24.6%−29.1%) compared with those in the pig farms located in the other two cities.

**Table 1:** The detection rate of PRRSV-1 in the clinical samples collected between 2020 and 2022 from pig farms in Xinjiang Uygur Autonomous Region.

**City**	**Farm**		**2020**			**2021**			**2022**		
			**Total**	**Blood**	**Lung**	**Total**	**Blood**	**Lung**	**Total**	**Blood**	**Lung**
Changji	FCH	Sample number	75	65	10	50	30	20	57	47	10
		Detection rate	66.7%	69.2%	50.0%	70%	66.7%	75.0%	50.9%	48.9%	60.0%
	YQ	Sample number	78	68	10	142	132	10	49	29	20
		Detection rate	50.0%	51.5%	40.0%	58.5%	56.8%	80.0%	55.1%	58.6%	50.0%
	JM	Sample number	79	69	10				88	78	10
		Detection rate	51.9%	50.7%	60.0%				62.5%	60.2%	80.0%
	ZS	Sample number				88	78	10	55	35	20
		Detection rate				32.9%	32.1%	40.0%	52.7%	51.4%	55.0%
	DB	Sample number				85	75	10	68	58	10
		Detection rate				58.8%	60.0%	50.0%	83.8%	81.0%	100%
Shihezi	142	Sample number				110	100	10	67	57	10
		Detection rate				68.2%	65.0%	100%	52.2%	52.6%	50.0%
	SZC	Sample number							75	65	10
		Detection rate							76.0%	75.3%	80.0%
Wujiaqu	105	Sample number							103	93	10
		Detection rate							29.1%	26.8%	50.0%
	SDB	Sample number							142	132	10
		Detection rate							24.6%	23.5%	40.0%

**Figure 1:**
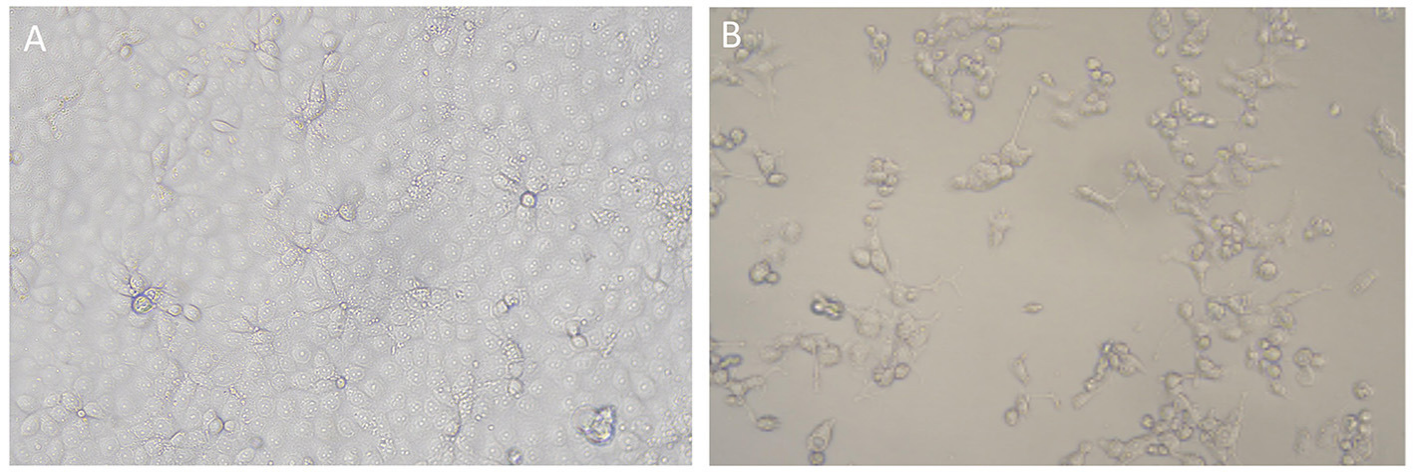
CPEs induced by PRRSV strain from Xinjiang in Marc-145 cells. **(A)** Mock cells; **(B)** Marc-145 cells infected by Xinjiang PRRSV strain, showing CPEs featuring cell shrinking, detachment, and vacuolization.

In addition, 100 PRRSV-positive blood or lung tissue samples were individually incubated with BMDM cell cultures before attempting adaptation to Marc-145 cells, in which most of the isolates failed to replicate upon passage, and CPE was not observed. However, 36 PRRSV strains were successfully adapted to rapid propagation in Marc-145 cells within 5 passages, with clear signs of CPEs featuring cell shrinking, fusion, cell layer splitting and vacuolization (Figure 1).

### 3.2 Phylogenetic analysis of PRRSVs in Xinjiang Uygur Autonomous Region

Meta-transcriptomics (MTT) technology was employed to perform high-throughput sequencing and genetic evolution analysis on 37 positive samples of porcine reproductive and respiratory syndrome virus (PRRSV) collected from nine large-scale farms. Reference strains were selected from various lineages of PRRSV1 and PRRSV2, including domestic and international strains, as well as six local strains of PRRSV2 from Xinjiang for analysis. The analysis results indicated that all 37 samples were successfully sequenced and identified as PRRSV2. The full-length of the viral genome ranged from 14,989 to 15,049 nucleotides (nts), which have been deposited in GenBank under accession numbers PP334602–PP334638 (Supplementary Table 1). Genome sequence analysis indicated that all 37 PRRSV strains identified here are grouped into sub-lineage 1.8 of PRRSV2 and share 86.6% nt sequence identity to sub-lineage 1.8 reference strain NADC30 (GenBank no.: JN654459) and 86.3% to sub-lineage 8.7 reference strain JXA1 respectively.

**Figure 2:**
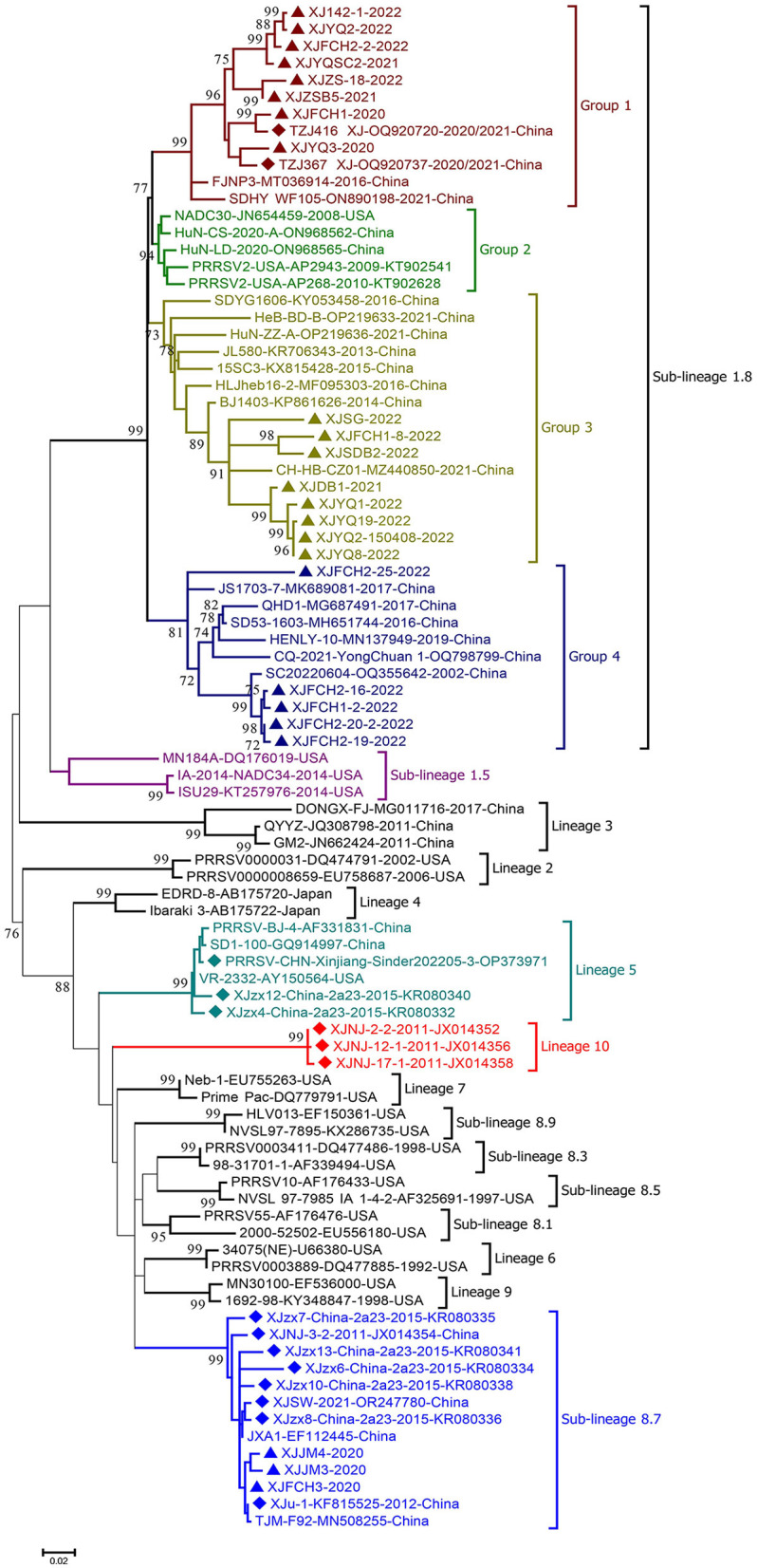
Phylogenetic tree based on the nucleotide sequences of ORF5 genes of PRRSV strains obtained in this study and reference strains. MEGA 7.0 was used for construction of the maximum-likelihood phylogenetic tree with bootstrap replicates of 1,000 and the best fitting substitution model of K2+G+I. Triangles: PRRSV strains obtained in the present study; diamonds: reference PRRSV strains from Xinjiang retrieved from GenBank.

Phylogenetic analysis of the complete ORF5 gene of the 37 strains with the sequences of 20 reference strains previously identified between 2011 and 2022 in Xinjiang showed that the new isolates classified into sub-lineages 1.8(34) and 8.7(3), indicating the dominant role of sub-lineage 1.8 in Xinjiang (Figure 2). All these PRRSV sub-lineage 1.8 strains shared 86.8%−100% nucleotide (nt) and 88.0%−100% amino acid (aa) sequence identities respectively, relatively close to the NADC30 strain (GenBank no.: JN654459). As shown in Figure 2, the sub-lineage 1.8 was further divided into 4 groups (Groups 1–4) with the nt and aa sequence identities between groups being 86.9%−95.9% and 87.5%−97.0% respectively. Group 2 consisted of NADC30 and its closely related strains, while the new strains in sub-lineage 1.8 classified into Groups 1, 3, and 4 with Group 1 collected from 4 farms (142, YQ, FCH, and ZS), Group 3 from 5 farms (SG, FCH, SDB, DB, and YQ), and Group 4 only from farm FCH. Of note is that 600 nt of ORF 5 of all Group 4 strains had a single codon deletion at aa 34 location in GP5 compared with reference strains. In addition, the 3 sub-lineage 8.7 strains were closely related to reference strains JXA1 and TJM-F92, sharing 98.5%−99.7% nt and 97.0%−99.0% aa sequence identity respectively. For the reference PRRSV strains collected between 2010 and 2022 from Xinjiang, strains belonging to sub-lineages 1.8 and 8.7, lineage 5 and a new lineage 10 were identified, indicating the genetic diversity of PRRSV strains circulating in the Xinjiang region. Among them, lineage 5 strains shared 99.0%−99.5% nt identity with modified attenuated live (MLV) vaccine strain VR-2332, and the sub-lineage 8.7 strains also most closely related to MLV vaccine strains JXA1 and TJM-F92 with a shared nt sequence identity of 96.8%−99.5%. The lineage 10 strains circulating in Xinjiang formed a separate clade from that formed by the known lineages 1–9 strains, showing 82.3%−90.4% nt sequence identity with other lineage strains. Further analysis of glycosylation sites in the GP5 protein demonstrated that glycosylation site at position 44 (NLT) is highly conserved among different genotype strains, while strains XJSDB2-2-2022, XJFCH2-16-2022 and XJFCH2-19-2022 have an additional potential glycosylation site at position 59 (NF/RS).

Multiple alignment of NSP2 protein sequences showed that, as compared to lineage 5 prototype strain VR-2332, all 37 PRRSV strains in this study had the same consensus 111+1+19 deletion pattern as the NADC30 strain. In addition, further deletions had occurred in some PRRSV strains. For strains XJFCH2-16-2022, XJFCH1-2-2022, XJFCH2-19-2022, XJFCH2-20-2022, 2 aa deletions were observed at sites 199–200. For XJYQ1-2020, XJYQ3-2020, and XJYQ2-2021, 2 aa deletions were present at sites 466–467. In PRRSV strain XJFCH2-25-2022, 3 aa deletions had occurred at sites 562-564. Of note is that the 3 sub-lineage 8.7 strains XJJM3-2020, XJJM4-2020, and XJFCH3-2020 showed the same deletion pattern in the NSP2 protein as sub-lineage 1.8 strains, indicating that recombination events had occurred in these strains.

### 3.3 Recombination events occurred in most PRRSV strains from Xinjiang

**Figure 3:**
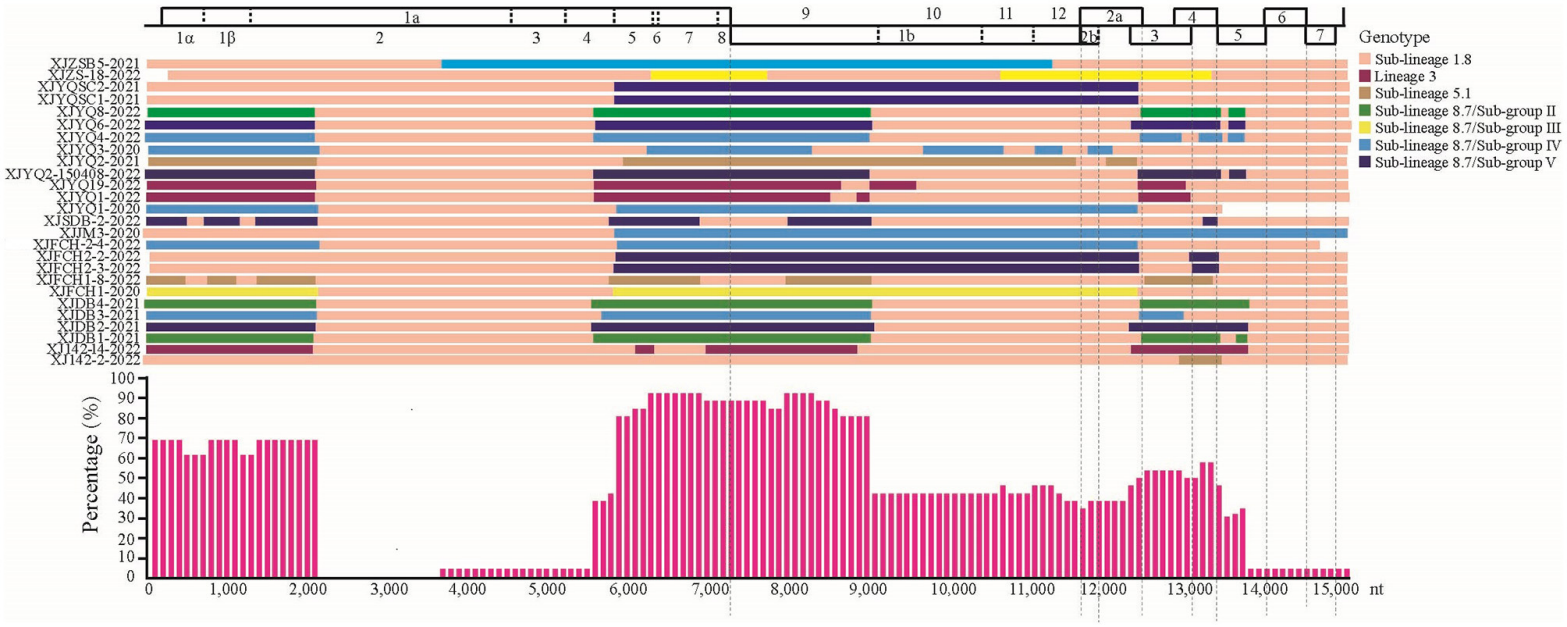
Maps of parental lineages of viral genomes and inter-lineage recombination patterns in PRRSV strains identified in this study. **Upper panel**: full-length genomic organization of reference strain NADC30. Dashes within the genomic organization graph indicate the positions and boundaries of the major ORFs and NSPs within ORF1 and ORF1b. Dashes under the graph were used to locate the range of ORFs. Different colors represented different PRRSV lineages. **Middle panel**: map of parental lineages of recombinant PRRSV strains identified in this study with the name of each strain displayed on the left, and with parental strains displayed in different colors according to the recombinant patterns. **Lower panel**: proportions of recombinationt events occurring in recombinant PRRSV strains. The X axis: PRRSV genome position; y axis: frequency of recombination that occurred at a specific region in a sliding window (per 100 nucleotide bases) centered on the position on the x axis.

Using RDP, recombinational events were found in 27 of the 37 PRRSV strains with 1–9 recombination domains in each strain (Table 2). Of note is that 22 recombinant Xinjiang PRRSV strains arose from the parental strains of sub-lineages 1.8 or 8.7 (Table 2), while recombination events between sub-lineage 1.8 and lineage 3 (3 events) or sub-lineage 5.1 (3 events) were also found, indicating easy recombination between sub-lineage 1.8 and sub-lineage 8.7 strains. As shown in Figure 3, most recombination events concentrated in the 5' and 3' termini of ORF1a, 5' terminus of ORF1b, and ORFs 3 and 4 of all Xinjiang strains (Figure 3).

**Table 2:** Recombination events occurred in the PRRSV strains collected from Xinjiang.

**Strain**	**Major parent**		**Minor parent**		**Recombination regions**
	**Strain**	**Lineage**	**Strain**	**Lineage**	
XJZSB5-2021	KP780881	Sub-lineage 8.7	KP860909	Sub-lineage 1.8	1
XJZS-18-2022	XJYQSC1-2021	Sub-lineage 1.8	KP742986	Sub-lineage 8.7	3
XJYQSC2-2021	MZ747439	Sub-lineage 1.8	FJ548853	Sub-lineage 8.7	2
XJYQSC1-2021	MZ747439	Sub-lineage 1.8	KC422727	Sub-lineage 8.7	2
XJYQ8-2022	JN654459	Sub-lineage 1.8	AY150312	Sub-lineage 8.7	3
XJYQ6-2022	KX766379	Sub-lineage 1.8	KX815422	Sub-lineage 8.7	1
XJYQ4-2022	JN654459	Sub-lineage 1.8	EF635006	Sub-lineage 8.7	2
XJYQ3-2020	XJYQSC1-2021	Sub-lineage 1.8	EU200962	Sub-lineage 8.7	1
XJYQ2-2021	MZ747439	Sub-lineage 1.8	GQ914997	Sub-lineage 5.1	3
XJYQ2-150408-2022	JN654459	Sub-lineage 1.8	AY150312	Sub-lineage 8.7	4
XJYQ19-2022	KX758249	Sub-lineage 1.8	MF124329	Lineage 3/QYYZ	3
XJYQ1-2022	KX758249	Sub-lineage 1.8	MF124329	Lineage 3/QYYZ	4
XJYQ1-2020	EU860249	Sub-lineage 8.7	MZ747439	Sub-lineage 1.8	2
XJSDB-2-2022	KR706343	Sub-lineage 1.8	MF669720	Sub-lineage 8.7	9
XJJM4-2020	EU860248	Sub-lineage 8.7	MZ747439	Sub-lineage 1.8	1
XJJM3-2020	EU860249	Sub-lineage 8.7	KP860909	Sub-lineage 1.8	2
XJJM3-2020	EU860248	Sub-lineage 8.7	MZ747439	Sub-lineage 1.8	2
XJFCH-84-2021	EF835006	Sub-lineage 8.7	MZ747439	Sub-lineage 1.8	2
XJFCH3-2020	KP742986	Sub-lineage 8.7	MZ747439	Sub-lineage 1.8	3
XJFCH2-3-2022	MZ747439	Sub-lineage 1.8	JQ804986	Sub-lineage 8.7	4
XJFCH2-2-2022	MZ747439	Sub-lineage 1.8	JQ804986	Sub-lineage 8.7	4
XJFCH1-8-2022	JN654459	Sub-lineage 1.8	KC445138	Sub-lineage 5.1	8
XJFCH1-2020	MF669720	Sub-lineage 8.7	MZ747439	Sub-lineage 1.8	3
XJDB4-2021	MN046225	Sub-lineage 1.8	AY150312	Sub-lineage 8.7	3
XJDB3-2021	KP860909	Sub-lineage 1.8	EF112447	Sub-lineage 8.7	3
XJDB2-2021	KX766379	Sub-lineage 1.8	KX815422	Sub-lineage 8.7	2
XJDB2-2021	JN654459	Sub-lineage 1.8	AY150312	Sub-lineage 8.7	3
XJDB1-2021	JN654459	Sub-lineage 1.8	JQ308798	Lineage 3/QYYZ	4
XJ142-2-2022	XJYQSC2-2021	Sub-lineage 1.8	GQ914997	Sub-lineage 5.1	1

## 4 Discussion

The Xinjiang Uygur Autonomous Region is located in north-western China and serves as the bridgehead for the initiative “One Belt, One Road” advocated by China, in which swine production is an important part of animal husbandry with more than 4 million pigs sold for slaughter annually. In Xinjiang, the convenient transportation of animals and animal products has also facilitated disease transmission, such as lumpy skin disease (Lu et al., 2021) and peste des petits ruminants (Wu et al., 2016), however there was less known about the prevalence of PRRS in the largest administrative region of China. The present work contributes to rectifying this situation. PRRSV infection in 9 farms in 3 main swine-producing cities in the Xinjiang Uygur Autonomous Region was monitored by collecting clinical PRRS-suspected samples between 2020 and 2022 for analysis of their genetic and evolutionary characteristics. Results showed that more than 50% of the swine farms were infected with PRRSV, especially those in Changji city, reflecting the severe epizootiologic situation of PRRSV in Xinjiang. Analysis of the isolated PRRSV strains indicated the dominant role of sub-lineage 1.8 between 2020 and 2022, which is also dominant in other regions of China; compared with other regions in China, however, the PRRSV strains circulated in this region were found to have a limited genetic diversity. An understanding of the dominant genotypes of PRRSV strains in this region is critical for developing scientific prevention and control strategies, such as positive serum determinations and development of safe and effective vaccines. While no PRRSV-1 strains were detected in the clinical samples analyzed here and collected between 2000 and 2022, they have been detected in samples collected in 2023 from several farms in this region (data not shown), and sub-lineage 1.5 (NADC34-like strains), another current dominant lineage in China and other countries was also not detected in the present study their very existence emphasizes the need for constant surveillance.

The spatial-temporal dynamics of PRRSV in Xinjiang showed that the dominant lineages of PRRSV strains circulated in different periods in this region vary: for instance, sub-lineage 8.7 was dominant in the 2010s, which then shifted to currently dominant sub-lineage 1.8, in which most strains are recombinant (Yu et al., 2017). Unexpectedly, a new lineage 10 was identified in PRRSV strains circulating in 2011 in southern Xinjiang, which formed a separate clade from other lineages in the phylogenetic tree (Figure 2), showing 9.6%−17.7% genetic distance from other lineage strains based on complete ORF5 gene sequences. Unfortunately, no other gene sequences of lineage 10 strains are available in GenBank to further establish the classification of this new lineage. Also noticed was that all Group 4 strains within sub-lineage 1.8 carry an aa deletion at site 34 of the GP5 protein, which is the biomarker for this group of PRRSV strains, although it remains unknown if this deletion alters the etiological properties. Although most sub-lineage 1.8 PRRSV strains from Xinjiang share the same deletion polymorphic patterns (111+1+19) of NSP2 protein as NADC30 (Yu et al., 2020), several strains have still additional deletions in this protein, further reflecting the genetic diversity of PRRSV strains.

In addition to point mutations and deletions, recombination serves as an important factor contributing to the rapid evolution of PRRSV strains. By analysis of recombination using RDP, more than 2/3 of the Xinjiang PRRSV strains identified in the present study were found to have arisen from recombination of 2 different lineage strains. Of note is that about 67% of the recombinant Xinjiang PRRSV strains arose from sub-lineages 1.8 and 8.7, the former being currently dominant in China, with the latter previously dominant, and MLV vaccines of this latter sub-lineage, including JXA1 and TJM-F92, were extensively used for vaccination of many pig herds, especially in the 2010s. In addition, recombination between sub-lineage 1.8 strains and another MLV vaccine, the VR-2332 strain, belonging to lineage 5 has also occurred in Xinjiang PRRSV strains. Based on the epizootiological situation of PRRS in China and the frequent recombination occurring in sub-lineage 1.8 strains, live vaccines cannot be considered a good choice for effective prevention and control of PRRS.

It has been reported that NADC30-like PRRSV strains (sub-lineage 1.8) were identified as moderately virulent strain through *in vivo* experiments (Brockmeier et al., 2012), while the virulence of this sub-lineage strain may be affected by viral recombination. For example, NADC30-like PRRSV strain FJ1402, which arose from recombination between the NADC30 and HP-PRRSV strains, has been demonstrated to show virulence similar to HP-PRRSV strain BB0907 (Zhang et al., 2016). Wang et al. (2018) reported that piglets injected with a recombinant NADC30-like PRRSV (SC-d), which was derived from recombination between JXA1-like and NADC30 strains, displayed higher pathogenicity than the non-recombinant NADC30-like PRRSV SD-A19, but lower than the HP-PRRSV HuN4 strain.

Overall, the present study has revealed the epizootiological situation and genetic diversity of PRRSV in Xinjiang, where sub-lineage 1.8 strains are currently dominant and mainly responsible for the circulation of PRRS in this region. Additionally, the recombinant sub-lineage 1.8 strains have frequently arisen from NADC30 strains, thereby demonstrating that MLV vaccine strains may not be the best choice for effective prevention and control of PRRS.

## Data availability statement

The genome sequences of Xinjiang PRRSV strains obtained in this study have been deposited in GenBank, and the related information can be found in the article/Supplementary material.

## Ethics statement

The animal studies were approved by the Ethics Committee of TECON Bio-pharmaceuticals Ltd. The studies were conducted in accordance with the local legislation and institutional requirements. Written informed consent was obtained from the owners for the participation of their animals in this study.

## Author contributions

JL: Writing – original draft, Conceptualization, Investigation, Resources, Funding acquisition, Project administration. WG: Writing – review & editing, Visualization. LM: Resources, Software, Visualization, Data curation, Writing – original draft. XP: Supervision, Data curation, Writing – review & editing. QW: Methodology, Resources, Writing – original draft. YG: Software, Methodology, Resources, Writing – original draft. JJ: Formal analysis, Software, Visualization, Writing – original draft. HT: Supervision, Formal analysis, Writing – review & editing. YZ: Resources, Writing – original draft. LC: Software, Writing – original draft. CT: Supervision, Writing – review & editing, Validation. XY: Conceptualization, Writing – review & editing, Validation. SH: Project administration, Supervision, Validation, Writing – review & editing. WZ: Conceptualization, Formal analysis, Software, Visualization, Writing – review & editing, Funding acquisition, Resources.
