# Supplementary material for: Molecular epizootiology of porcine reproductive and respiratory syndrome virus in the Xinjiang Uygur Autonomous Region of China
Source: Front Microbiol. 2024 Jun 26;15:1419499. doi: 10.3389/fmicb.2024.1419499 (PMC11233727; doi:10.3389/fmicb.2024.1419499)
Supplement: Supplementary file 1 [file Data_Sheet_1.docx]

Supplemental table 1 GenBank accession numbers for the complete genome sequences of 37 PRRSV isolates from Xinjiang

| PRRSV isolates | Lineage | GenBank Accession No. |
| --- | --- | --- |
| XJZSB5-2021 | sub-lineage 1.8 | PP334602 |
| XJZS-18-2022 | sub-lineage 1.8 | PP334603 |
| XJYQSC2-2021 | sub-lineage 1.8 | PP334604 |
| XJYQSC1-2021 | sub-lineage 1.8 | PP334605 |
| XJYQ19-2022 | sub-lineage 1.8 | PP334606 |
| XJYQ8-2022 | sub-lineage 1.8 | PP334607 |
| XJYQ6-2022 | sub-lineage 1.8 | PP334608 |
| XJYQ4-2022 | sub-lineage 1.8 | PP334609 |
| XJYQ3-2020 | sub-lineage 1.8 | PP334610 |
| XJYQ2-150408-2022 | sub-lineage 1.8 | PP334611 |
| XJYQ2-2022 | sub-lineage 1.8 | PP334612 |
| XJYQ2-2021 | sub-lineage 1.8 | PP334613 |
| XJYQ1-2022 | sub-lineage 1.8 | PP334614 |
| XJYQ1-2020 | sub-lineage 1.8 | PP334615 |
| XJSG1-2022 | sub-lineage 1.8 | PP334616 |
| XJSDB-2-2022 | sub-lineage 1.8 | PP334617 |
| XJJM4-2020 | sub-lineage 8.7 | PP334618 |
| XJJM3-2020 | sub-lineage 8.7 | PP334619 |
| XJFCH3-2020 | sub-lineage 8.7 | PP334620 |
| XJFCH2-25-2022 | sub-lineage 1.8 | PP334621 |
| XJFCH2-20-2022-2 | sub-lineage 1.8 | PP334622 |
| XJFCH2-20-2022 | sub-lineage 1.8 | PP334623 |
| XJFCH2-19-2022 | sub-lineage 1.8 | PP334624 |
| XJFCH2-16-2022 | sub-lineage 1.8 | PP334625 |
| XJFCH2-3-2022 | sub-lineage 1.8 | PP334626 |
| XJFCH2-2-2022 | sub-lineage 1.8 | PP334627 |
| XJFCH2-2-4-2022 | sub-lineage 1.8 | PP334628 |
| XJFCH1-2020 | sub-lineage 1.8 | PP334629 |
| XJFCH1-8-2022 | sub-lineage 1.8 | PP334630 |
| XJFCH1-2-2022 | sub-lineage 1.8 | PP334631 |
| XJDB4-2021 | sub-lineage 1.8 | PP334632 |
| XJDB3-2021 | sub-lineage 1.8 | PP334633 |
| XJDB2-2021 | sub-lineage 1.8 | PP334634 |
| XJDB1-2021 | sub-lineage 1.8 | PP334635 |
| XJ142-14-2022 | sub-lineage 1.8 | PP334636 |
| XJ142-6-2022 | sub-lineage 1.8 | PP334637 |
| XJ142-2-2022 | sub-lineage 1.8 | PP334638 |
